# Virus-triggered exacerbation in allergic asthmatic children: neutrophilic airway inflammation and alteration of virus sensors characterize a subgroup of patients

**DOI:** 10.1186/s12931-017-0672-0

**Published:** 2017-11-14

**Authors:** Antoine Deschildre, Muriel Pichavant, Ilka Engelmann, Carole Langlois, Elodie Drumez, Guillaume Pouessel, Sophie Boileau, David Romero-Cubero, Irina Decleyre-Badiu, Anny Dewilde, Didier Hober, Véronique Néve, Caroline Thumerelle, Stéphanie Lejeune, Clémence Mordacq, Philippe Gosset

**Affiliations:** 1 University Lille, U1019 - UMR 8204 - CIIL - Center for Infection and Immunity of Lille, F-59000 Lille, France; 2 CNRS, UMR 8204, F-59000 Lille, France; 3 Inserm, U1019, F-59000 Lille, France; 4 CHU Lille, F-59000 Lille, France; 5 Institut Pasteur de Lille, F-59000 Lille, France; 6 CHU Lille, Unité de Pneumologie et Allergologie Pédiatrique, Hopital Jeanne de Flandre, F-59000 Lille, France; 7 CHU Lille, Service de Virologie, F-59000 Lille, France; 8 University Lille, EA 3610 - Pathogenèse virale du diabète de type 1, F-59000 Lille, France; 9 CHU Lille, Departement de Biostatistiques, F-59000 Lille, France; 10 University Lille, EA 2694 - Santé publique: épidémiologie et qualité des soins, Département de Biostatistique, F-59000 Lille, France; 11 CH Roubaix, Service de Pédiatrie, Hôpital Victor Provo, F-59100 Roubaix, France; 12 CHU Lille, Service d’Exploration Fonctionnelle Respiratoire, F-5900 Lille, France; 13 INSERM U1019-CNRS UMR8204, CIIL, “Lung infection and innate immunity” research group, Institut Pasteur de Lille, 1 Rue du Professeur Calmette, F-59019 Lille cedex, France

**Keywords:** Allergic asthma, Exacerbation, Viral infection, Pattern recognition receptor, Interferon

## Abstract

**Background:**

Viruses are important triggers of asthma exacerbations. They are also detected outside of exacerbation. Alteration of anti-viral response in asthmatic patients has been shown although the mechanisms responsible for this defect remain unclear. The objective of this study was to compare in virus-infected and not-infected allergic asthmatic children, aged 6 to 16 years, admitted to hospital for a severe exacerbation, the innate immune response and especially the expression of pattern recognition receptor (PRR) and their function.

**Methods:**

Virus identification was performed both during the exacerbation and at steady state (eight weeks later). Data assessed at both periods included clinical features, anti-viral response and inflammation (in sputum and plasma), and PRR expression/function in blood mononuclear cells.

**Results:**

Viruses were identified in 46 out of 72 children (median age 8.9 years) during exacerbation, and among them, in 17 at steady state. IFN-β, IFN-γ and IL-29 levels in sputum and plasma were similar between infected and not infected patients at both times, as well as the expression of TLR3, RIG-I and MDA5 in blood monocytes and dendritic cells. Airway inflammation in infected patients was characterized by significantly higher IL-5 concentration and eosinophil count. Compared to patients only infected at exacerbation, the re-infected children significantly exhibited lower levels of IFN-γ in plasma and sputum at exacerbation associated with modifications in PRR expression and function in blood mononuclear cells. These re-infected patients also presented an airway neutrophilic inflammation at steady state.

**Conclusion:**

Our results reports in asthmatic children that impaired anti-viral response during virus-induced exacerbation is more pronounced in a subgroup of patients prone to re-infection by virus. This subgroup is characterized by altered PRR function and a different pattern of airway inflammation.

**Trial registration:**

This multicenter prospective study was approved by the regional investigational review board (ref: 08/07).

**Electronic supplementary material:**

The online version of this article (10.1186/s12931-017-0672-0) contains supplementary material, which is available to authorized users.

## Background

Respiratory viruses, mainly human rhinoviruses (hRV) are major triggers of exacerbation in asthmatic children [1, 2]. Viruses first target airway epithelial cells (AEC) and then antigen-presenting cells (APC), including conventional and plasmacytoid dendritic cells (cDC and pDC, respectively), via the mobilization of pattern recognition receptors (PRR), such as toll-like receptors (TLR) and RNA helicases (RIG-I, MDA5) [3, 4]. hRV induce innate interferon (IFN) production in AEC via RIG-I, MDA5 and TLR3 [5, 6] whereas influenza virus requires TLR7 [7]. Impairment in anti-viral response has been reported in asthmatic patients infected with hRV, as shown by altered production of type I IFNs (IFN-α/β) and/or type III IFNs [interleukin (IL)-28 and IL-29] [8–11]. However, if the deficient IFNs response has been reported in severe asthma, associated with a defect in TLR activation, it was not observed in well controlled asthma [12–14].

To our knowledge, no data are available regarding the expression and function of PRR during exacerbation. We hypothesized that alteration of the expression and/or function of virus sensors is associated with impaired innate immune response during virus-induced asthma exacerbation. Moreover, these alterations might impact on clinical and inflammatory profiles. To test this hypothesis, the anti-viral response and the expression and function of the virus sensors in blood mononuclear cells were explored in a cohort of allergic asthmatic children admitted to hospital with a diagnosis of severe exacerbation. Evaluation also included clinical features and airway and blood inflammation and was done at exacerbation and repeated at steady state, 8 weeks later. First, we compared virus infected to not-infected patients at exacerbation. Secondly, as our results showed that an infection with a different virus is frequently detected in asthmatic patients at steady state [15, 16], we focused on the virus-infected patients at exacerbation in order to compare patients infected at both times to those only infected at the exacerbation.

Our data demonstrated that impairment of IFN production and virus sensor function was mainly observed in the subgroup of asthmatic children re-infected at steady state.

## Methods

### Study design and patients

This multicenter prospective study, approved by the regional investigational review board (Comité de protection des personnes Nord Ouest, ref.: 08/07) involved the Pediatrics Departments of Lille University Hospital (Lille, France) and Roubaix Hospital (Roubaix, France). Parental written informed consents were obtained for all children.

Children aged between 6 and 16 years with a diagnosis of allergic asthma who were admitted to hospital for a severe exacerbation were eligible for inclusion. The severity of the exacerbation was assessed according to the guidelines [17]. All the patients were treated with systemic corticosteroids. Allergic sensitisation was defined by at least one allergen-specific IgE ≥ 0.35 kUA/L and/or or a positive skin prick test. Exclusion criteria were congenital or acquired chronic illnesses other than asthma.

### Study protocol and outcomes

Subjects were assessed twice: at exacerbation during hospitalization and at steady state during a follow-up visit scheduled 8 weeks later (±1 week).

Baseline characteristics including demographic characteristics, personal comorbidities (allergic rhinitis, atopic dermatitis, food allergy), history of asthma exacerbations and passive tobacco exposure were recorded. Maintenance treatment was documented and inhaled corticosteroid dose was expressed in fluticasone equivalent μg per day (μg/d). The lengths of the oxygenotherapy (days) and of the hospitalization (days) were collected.

Viral status, local (sputum) and systemic IFN response and inflammatory reaction (cytokines; sputum inflammatory cell counts), and PRR expression and function were studied at both times.

At steady state, asthma control was evaluated and spirometry was performed. Asthma control was assessed according to GINA criteria (well controlled, partially controlled or uncontrolled) (www.ginasthma.com). Spirometry and bronchodilator reversibility were measured according to American Thoracic Society and European Respiratory Society Recommendations [18]. Forced vital capacity (FVC) and FEV1 were expressed in percentage of predicted value (%VP), FEV1 / FVC in absolute value. Exhaled nitric oxide (eNO) was also measured and expressed in ppb [18, 19].

Subjects were first grouped and compared according to viral infection at exacerbation: infected (V+) and not-infected (V-) patients. Following the description of patients who were infected at both exacerbation and steady state (V + V+ patients), we compared this subgroup to the patients only infected at the exacerbation (V + V- patients) [15]. The low number of patients infected at steady state among the V- patients did not allow studying this subgroup.

### Blood and sputum collection

Spontaneous or induced sputum, peripheral blood mononuclear cells (PBMC) and plasma were collected at exacerbation (first 2 days) and at steady state. Plasma from blood samples was used to measure cytokine concentrations. Blood mononuclear cells (MNC) were isolated using a Ficoll-Paque density gradient. After washings, cells were resuspended in RPMI 1640 supplemented with 10% heat-inactivated fetal calf serum and antibiotics (Life technologies) or with PBS with 2% heat-inactivated fetal calf serum for cell culture or flow cytometry, respectively. Isolated MNC were stimulated with a ligand for TLR3: synthetic double-stranted RNA (poly(IC)) (5 μg/ml), a ligand for RNA-helicases: liposome-polyIC (lipoP(I:C), 2 μg/ml), a ligand for TLR7-8: Guardiquimod (2 μg/ml), and phytohemagglutinin (PHA) as a positive control (Invivogen, San Diego, Ca). Supernatants were collected after 24 h of culture.

Induced sputum samples were collected after nebulization of isotonic (at exacerbation) or hypertonic (steady state) saline solution as previously described [20–22]. Plugs were isolated from the sputum, weighted and processed as previously described [20]. Briefly, plugs were diluted with sputolysin (VWR) and then, sputum fluids and cells were separated by centrifugation. The isolated cells were used for differential cell counts and the fluid for cytokine measurements. Cytospins were prepared from the cell pellets and the supernatants were stored at −80 °C. Samples with more than 30% of squamous cells were excluded from further analysis and differential leukocyte cell counts were undertaken by counting 300 non-squamous cells in sputum samples.

### Virus identification

Nasal secretions were collected for each patient at inclusion (exacerbation) and at steady state. Samples were frozen (−80 °C) before RNA extraction. A commercially available multiplex reverse transcription–polymerase chain reaction (RT-PCR) screened 15 respiratory viral pathogens including influenza virus A and B, respiratory syncytial virus A and B, adenovirus, metapneumovirus, coronavirus 229E/NL63 and OC43, parainfluenza virus 1–4, rhinovirus A/B/C, enterovirus, and bocavirus 1–4 (Seeplex RV15 ACE Detection, Seegene, Seoul, Korea). Specimens with detection of rhinovirus were typed by amplification and sequencing of the viral protein (VP) 4/VP2 region using the primers described by Wisdom et al. [23].

Quantitation of HRV RNA was performed according to Tapparel et al. [24, 25]. Briefly, one step real-time RT PCR was performed using the QuantiTect probe RT-PCR kit (Qiagen) and the primers and probes: AGCCTGCGTGGCKGCC, CYlnaAGCClnaTGCGTGG, FAM-CTCCGGCCCCTGAATGYGGCTAA-TAMRA, GAAACACGGACACCCAAAGTAGT. Reactions were run on a TaqMan 7500 (Applied Biosystems) thermocycler under the following cycling conditions: 50 °C for 30 min, 95 °C for 15 min and 45 cycles of 94 °C for 15 s and 60 °C for 1 min.

Rhinovirus A9 was propagated on MRC5 cells and supernatant was quantified in TCID50/mL. RNA of culture supernatant was extracted and serial 10-fold dilutions submitted to the quantitative RT PCR in order to establish a standard curve for quantification. Results of quantification are expressed as TCID50/mL equivalents.

### Flow cytometry

To analyze the activation and the expression of PRR (TLR3, MDA5 and RIG-I) within blood DC and monocytes, PBMC were incubated for 30 min on ice with isotype-matched control antibodies for lymphocytes and granulocytes (lin-1), DC (HLA-DR, CD11c, CD123 and CD86) and monocytes (CD14). Monocytes were defined as CD14^+^ cells. cDC and pDC subsets were respectively defined by the Lineage1^−^ CD14^−^ HLA-DR^+^ CD123^+^ and Lineage1^−^ CD14^−^CD11c^+^ HLA-DR^+^ phenotypes as illustrated in the Additional file 1. Cell activation in APC was analyzed by measurement of the median of fluorescence (MFI) for HLA-DR and CD86 (BD-Biosciences). Moreover, the expression of TLR3, RIGI and MDA5 (Santa-Cruz Biotechnology) was estimated by indirect labeling after cell permeabilization. A corresponding isotype control was included to define the background level and the results were expressed after subtraction of the value obtained with the isotype control.

### Cytokine assays

PBMC were stimulated with ligands for TLR3 [polyinosinic:polycytidylic acid, poly(I:C)], RNA-helicases [poly(I:C) liposome, lipopoly(I:C)] and TLR7-8 (gardiquimod) (InvivoGen, San Diego, CA). PBMC supernatants were collected at baseline and 24 h after stimulation. Levels of IL-4, IL-5 (Th2 cytokines), CXCL8, IL-17, IL-22 (R&D Systems, Abingdon, UK), IFN-γ, IL-1β, IL-6, IL-29 (IFN-λ) (eBiosciences, San Diego, CA) and IFN-β (Elabsciences Biot., Wuhan, China) in plasma, sputum fluids and supernatants from PBMC were measured by ELISA. On the whole population, P(I:C) increased the secretion of IL-4, IL-5, IL-6, IL-29, IFN-β, IFN-γ and CXCL8 as compared to cells in medium alone, whereas gardiquimod and lipoP(I:C) upregulated the levels of IL-1β, IL-6, IFN-β, IFN-γ and CXCL8 (data not shown).

### Statistical analysis

Statistical analyses were performed by SAS 9.3 software (SAS Institute Inc., Cary, NC 25513). Qualitative variables are reported as the number or the percentage and compared via the chi-square test or Fisher’s exact test. Continuous variables, reported as median [interquartile range (IQR)], were compared via the Mann-Whitney test. Normality was assessed via the Shapiro-Wilk test. A *p*-value <0.05 was considered to be statistically significant. To evaluate the magnitude of differences between groups, we calculated the absolute standardized differences; a standardized difference between 20 and 50, 50 and 80 and higher than 80% denotes low, medium and large imbalance, respectively [26].

First, V+ were compared to V- patients at exacerbation and at steady state and in a second step, V+ V+ were compared to V + V- patients. Then, exacerbation conditions were compared to Steady state in each group: V+, V-, V + V- and V + V+.

## Results

### Patients

**Fig. 1:**
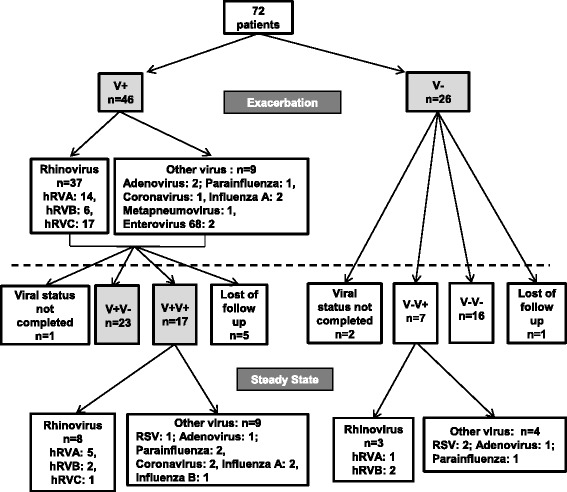
The viral status of the population during the exacerbation and at steady state. hRV: human rhinovirus, RSV: respiratory syncytial virus; V+: viral infection; V-: no detected virus. In this study, we first compared infected and not-infected groups (V+ versus V- patients, shaded boxes) and then among V + V- and V + V+ patients

Seventy-two patients (median age 8.9 years [IQR: 7·7-11·7]; boys: 73%) were included (Table 1) among which 32 (43%) were under a maintenance treatment at inclusion. A virus was detected in 46 patients (62%), hRV in 37 of them (Fig. 1). Median hRV load was 2341 TCID50/mL equivalents [1492 – 18,689]. Clinical features of the exacerbation were similar in V+ and V- patients (Table 1 and see Additional file 2).

**Table 1:** Description of the study population and characteristics of the exacerbation, according to the viral status at exacerbation and at steady state

	All	V+	V-	V + V+	V + V-	V+ versus V- *P* value (ASD)	V + V+ versus V + V- *P* value (ASD)
Total (n)	72	46	26	17	23		
Median age (years)	8.9 [6–15.3]	9.2 [6–15]	8.7 [6.2–15.3]	8.4 [6–12]	9.8 [6–14,3]	0.35 (22.6)	0.04 (71.9)
Gender							
- Boys (n)	52	31	21	13	15	0.22	0.44
- Girls (n)	20	15	5	4	8	(30.9)	(25.0)
Frequent exacerbations (>2/y) (n)	27	18	9	6	12	0.70 (9.4)	0.29 (34.5)
Maintenance treatment before inclusion (n)	32	21	11	ND	ND	0.78 (6.7)	0.96 (1.5)
Atopic dermatitis (n)	30	16	14	5	9	0.11 (39.1)	0.51 (20.6)
Allergic rhinitis (n)	50	31	19	11	15	0.61 (12.5)	0.97 (1.1)
Food allergy (n)	11	6	5	2	3	0. 51 (16.9)	1 (3.9)
Passive tobacco exposure (n)	39	23	16	9	12	0.39 (21.1)	0.96 (1.5)

*V+* identification of viral infection (PCR) at inclusion, *V–* no identification of viral infection at inclusion, *V + V+* identification of viral infection at inclusion and at the steady state, *V + V–* identification of a viral infection at inclusion but not at the steady state, *ND* not done, *ASD* absolute standardized difference (%)

Results were expressed as numbers and medians with interquartile range between brackets or

Sixty-six patients (91%) were evaluated at steady state. None had symptoms of exacerbation. According to GINA, asthma was well controlled in 22 patients (33%) (Table 2). A virus was identified in 24 patients (36%), including 7 without infection during exacerbation (Fig. 1). Different viruses were detected at exacerbation and at steady state in 17 V + V+ patients (24%), as previously reported [15]. Moreover, the repartition of the inclusions during the year was not different among V+ and V- patients as well as for V + V- and V + V+ patients [see Additional file 3].

**Table 2:** Asthma control and lung function at steady state, according to the viral status at exacerbation and steady state

	All	V+	V-	V + V+	V + V-	*p* value (ASD)	*p* value (ASD)
	*n* = 66	*n* = 41	*n* = 25	*n* = 17	*n* = 23	V+ versus V-	V + V+ versus V + V-
Controlled asthma (GINA) (n)	22	14	8	7	7	0.86 (4.6)	0.48 (22.6)
ACT or C-ACT* median [IQR]	22 [20–25]	22 [20–25]	22 [19–25]	23 [21–25]	21 [20–24]	0.58 (13.9)	0.18 (43.8)
Lung function							
-FEV1 (% of PV) Pre β2 agonist [IQR]	96 [86–108]	97 [86–109]	96 [85–108]	94 [86–108]	98 [86–110]	0.51 (12.5)	0.79 (7.7)
-FEV1 (% of PV)Post β2 agonist [IQR]	109 [99–117]	108 [97–116]	113 [100–118]	108 [100–115]	108 [95–119]	0.22 (5.7)	0.82 (15.5)
-FEV1/FVC (%)Pre β2 agonist [IQR]	81 [76–86]	82 [77–86]	81 [75–86]	84 [76–89]	79 [77–84]	0.46 (19.5)	0.16 (43.9)
-FEV1/FVC (%) Post β2 agonist [IQR]	88 [83–91]	89 [84–92]	87 [82–89]	90 [86–93]	89 [82–91]	0.23 (0.2)	0.21 (48.2)
-eNO (ppb) [IQR]	17 [10–32]	22 [11–46]	13 [9–22]	22 [13–27]	23 [11–47]	0.06 (51.2)	0.62 (7.8)

*V+* identification of a virus (PCR) at exacerbation, *V–* no identification of a virus at exacerbation, *V + V+* identification of a virus at exacerbation and steady state, *V + V–* identification of a viral infection at exacerbation but not at steady state. Results were expressed as numbers or medians with interquartile range between brackets. *ASD* absolute standardized difference (%)

### Comparison of the immune responses among V+ and V- patients

#### Production of IFNs

**Fig. 2:**
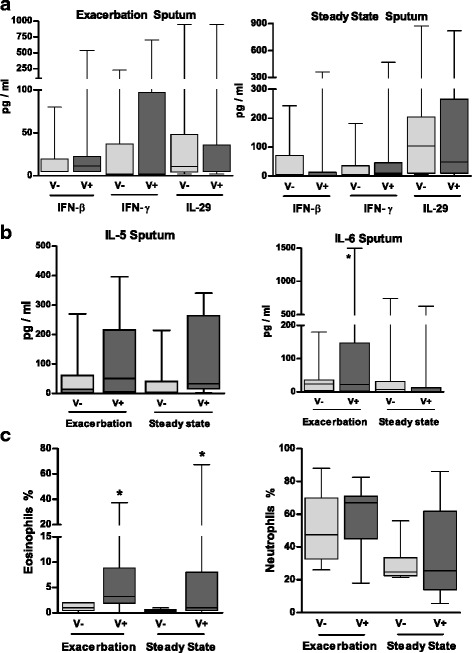
Impact of the viral status on cytokine concentrations and polymorphonuclear cells in sputum from asthmatic children collected during exacerbation and at steady state. Concentrations of IFN-β, IFN-γ and IL-29 (**a**) and of IL-5 and IL-6 (**b**) were measured in the sputum fluids collected from infected patients (V+) and non-infected patients (V-) during asthma exacerbation and at steady state. **c**) The percentages of eosinophils and neutrophils in the sputum are also reported. The median of our results was reported by the horizontal line, the 25 and 75 interquartile range by the box and the minimum and the maximum by the whiskers. *: *p* < 0.05 versus V- patients

Similar levels of IFN-β, IL-29 and IFN-γ were detected in the sputum (Fig. 2a) and in the plasma [see Additional file 2] of V+ and V- patients during exacerbation and at steady state.

#### Characteristics of airway and blood inflammation

At both times, V+ patients had significantly higher IL-5 levels in sputum (Fig. 2b) and plasma [see Additional file 4] than in V- patients, these levels being associated with higher percentages of sputum eosinophils (*p* < 0·05) (Fig. 2c). Eosinophil percentages were significantly correlated with the levels of IL-5 in sputum (*r* = 0·66, *p* < 0·005) but not in plasma (r = 0·47, p = NS). In contrast, sputum neutrophil percentages and numbers were not different between V+ and V- (Fig. 2c and Table 3). During exacerbation only, IL-6 levels were significantly greater in sputum (Fig. 2b) and plasma of V+ patients [see Additional file 4]. A trend towards increased CXCL8 levels in sputum was also observed. Plasma IL-22 levels were significantly lower in V+ patients at steady state whereas the levels of the other cytokines, including IL-17 and IL-4 were not different [see Additional file 4 and data not shown].

**Table 3:** Results of cell analyses in induced sputum

*Exacerbation*	*V+*	*V-*	*V + V+*	*V + V-*
Patients (N)	33	10	9	20
Sputum weight (mg)	365 [201–560]	330.5 [90–710]	490 [460–575]	275 [128–630]
n ×10^3^ total cells/mg	6 [3.4–16.1]	13.6 [7.8–24.5]	10 [3.5–20]	5.5 [2.8–11.1]
Epithelial cells (%)	14 [8–24]	11.7 [5–14]	8 [6–12]	20 [11–24.5]
Neutrophils (%)	68 [45.5–71]	47.5 [33–65.5]	68 [31–69.5]	65.5 [45.2–69.7]
Eosinophils (%)	3.5 **[2–8.5]	1 [0.5–2]	2 [1–14.5]	4.25 [2.3–9]
Macrophages (%)	10 * [7–21]	32 [9–51]	10 [8–21]	12 [6.5–21]
Lymphocytes (%)	1 [0.5–2]	2 [1–3]	1 [0.5–1.5]	1 [0.5–2]
*Steady state*				
Patients (N)	16	5	7	8
Sputum weight (mg)	200 [85–325]	320 [15–330]	240 [40–420]	200 [135–260]
n ×10^3^ total cells/mg	3.5 [2.2–15.6]	6.67 [3.1–9.4]	16.7 [2–24]	2.4 [2.1–3.5]
Epithelial cells (%)	22 [13–35]	48 [8–66]	21 [6–28]	34.5 [20.5–50.3]
Neutrophils (%)	26 [14–57.5]	25.5 [21.5–56]	57.5 ^#^[34–81]	16.3 [10.5–39]
Eosinophils (%)	1 *[0.5–8]	0.5 [0–0.5]	1 [0.5–26]	1.5 [0.5–8]
Macrophage (%)	12 [6–38]	23.5 [10–30.5]	9 [5–11]	27.5 [9–38.5]
Lymphocytes (%)	1 [0.5–2]	3 [2–5]	1 [0.5–2]	2.5 [1–5.5]

Patients were evaluated during the exacerbation and at steady state. Results were expressed as numbers and medians with interquartile range between brackets. Differences were considered as statistically significant after analysis by Mann–Whitney (*: *p* < 0.05; **: *p* < 0.01 versus V–) (^#^: *p* < 0.05 versus V + V–). ASD: absolute standardized difference (%)

Compared to steady state, only CXCL8 and IL-6 levels in the sputum of V+ patients were significantly higher during exacerbation [see Additional file 4].

#### TLR expression and function in PBMC

**Fig. 3:**
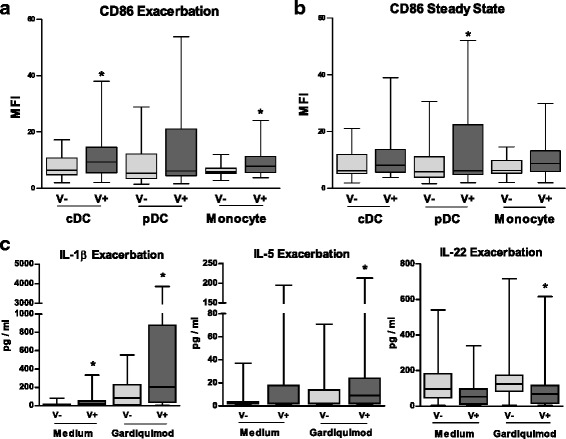
Impact of viral status on blood mononuclear cell phenotype during asthma exacerbation and at steady state. **a**-**b**) CD86 expression was measured on circulating cDCs, pDCs and monocytes from asthmatic children during the exacerbation (**a**) and steady state (**b**), using flow cytometry. During the exacerbation, patients were classified as being infected (V+) or not infected (V-). **c**) The levels of IL-6, IL-5 and IL-22 were measured in supernatants of PBMCs incubated in medium alone (baseline) and then stimulated with the TLR7-8 ligand gardiquimod. The median of our results was reported by the horizontal line, the 25 and 75 interquartile range by the box and the minimum and the maximum by the whiskers. *: *p* < 0.05 versus V- patients

Expression levels of TLR3, RIG-I and MDA5 by blood DC and monocytes were similar in V+ and V- patients at both times [see Additional file 5]. Expression of the costimulatory molecule CD86 was greater in cDC and monocytes of V+ than V- patients during exacerbation and in pDC at steady state (*p* < 0·05, Fig. 3a-b). In contrast, HLA-DR levels were similar.

Compared to V-, IL-1β was increased in unstimulated PBMC of V+ (*p* < 0·05) [see Additional file 4]. In response to PRR ligands, IL-29 levels were higher in gardiquimod (TLR7-8 ligand)-stimulated PBMC from V+ patients at both time points whereas IFN-β and IFN-γ levels remained similar whatever the stimulus [see Additional file 4]. During exacerbation only, IL-1β and IL-5 levels were greater in gardiquimod-stimulated PBMC among V+ patients (*p* < 0.05), whereas IL-22 levels were lower (*p* < 0·05) (Fig. 3c). At steady state, IL-1β and IL-6 levels were higher in poly(I:C) (TLR3 ligand)-stimulated PBMC among V+ patients (*p* < 0·05) [see Additional file 4].

Compared to the steady state, the levels of IFN-γ, IL-1β and IL-6 at exacerbation were lower in unstimulated and poly(I:C)-stimulated PBMC among V+ and V- patients [see Additional file 4]. IL-5 production by unstimulated PBMC from V+ patients and IL-1β secretion in gardiquimod-stimulated PBMC were also lower (*p* < 0.05). The other cytokines did not change.

### Characteristic features of the immune response and the PRR in V + V+ patients

#### Production of IFNs

**Fig. 4:**
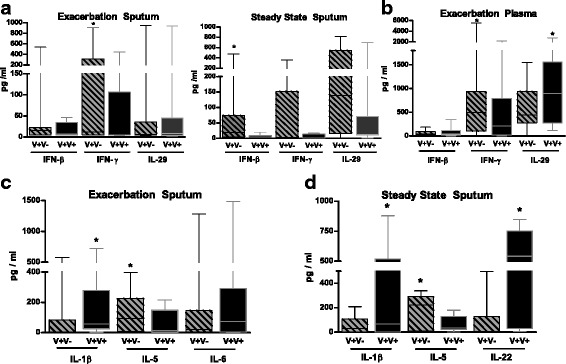
Viral re-infection in asthmatic patients modulates cytokine secretion. **a**) IFN-β, IFNγ and IL-29 concentrations were measured in the sputum fluids and the plasma (B) collected during the exacerbation and at steady state in V+ patients separated in re-infected ones (V + V+) and not-infected ones at steady state (V + V-). **b**) Concentrations of IFN-β, IFNγ and IL-29 were analyzed in the plasma collected at exacerbation in both groups. **c**) Levels of IL-1β, IL-5 and IL-6 were evaluated in the sputum fluids collected during the exacerbation from the same groups. **d**) Concentrations of IL-1β, IL-5 and IL-22 were also reported in the sputum fluids collected at steady state. The median of our results was reported by the horizontal line, the 25 and 75 interquartile range by the box and the minimum and the maximum by the whiskers. *: *p* < 0.05 versus V+V- patients

Compared to V + V- patients, sputum IFN-γ levels (*p* < 0.05) at exacerbation were lower in V + V+ patients, with no significant change for IFN-β and IL-29 (Fig. 4a). In plasma, IFN-γ levels were lower in V + V+ whereas the IL-29 concentrations were greater (*p* < 0.05) and IFN-β levels did not differ (Fig. 4b). At steady state, levels of IFN-β (*p* < 0.05) and IL-29 (p = NS) were lower in V + V+ patients in sputum (Fig. 4a). Concentrations of blood IFNs were similar between V + V+ and V + V- patients at steady state [see Additional file 6].

#### Characteristics of airway and blood inflammation

Sputum IL-5 concentrations were lower in V + V+ than in V + V- patients at both periods (*p* < 0.05) (Fig. 4c) whereas eosinophils did not differ. In contrast, the percentages of neutrophils were significantly higher in V + V+ patients at steady state (*p* < 0.05) (Table 3). During exacerbation and at steady state, sputum concentrations of IL-1β (*p* < 0.05) were higher in V + V+ than in V + V- patients (Fig. 4c). Higher IL-22 levels were detected in sputum of V + V+ patients at steady state (*p* < 0.05) (Fig. 4d). Finally, plasma IL-6 concentrations were greater in V + V+ than V + V- patients during exacerbation (*p* < 0.05) whereas there were no differences for the other cytokines both in sputum and plasma [see Additional file 6].

The comparison between steady state and exacerbation among V + V+ and V + V- groups revealed that plasma and sputum cytokine levels did not differ.

#### TLR expression and function in PBMC

**Fig. 5:**
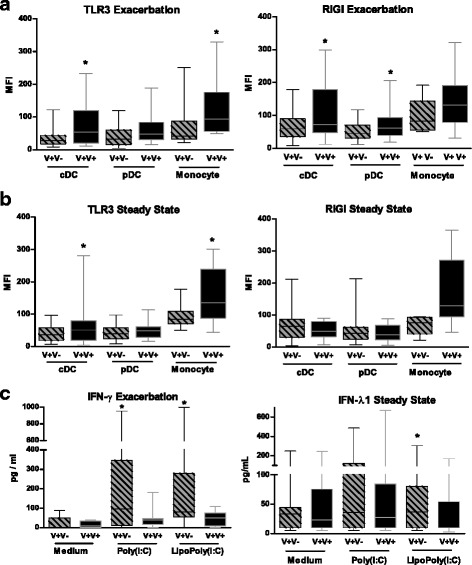
Viral re-infection in asthmatic patients modulate blood mononuclear cell phenotype and cytokine secretion. **a**) TLR3 expression was measured by flow cytometry on cDC, pDC and monocytes studied during the exacerbation and at steady state. Blood cells were collected from V+ patients separated in re-infected ones (V + V+) and not-infected ones at steady state (V + V-). **b**) The fluorescence intensity for RIGI was also evaluated in these cells studied during the exacerbation and at steady state. **c**) Concentrations of IFN-γ and IL-29 were measured in supernatants of PBMC collected in V + V+ and V + V- patients during the exacerbation and at steady state. PBMC were incubated in medium alone and stimulated with Poly(I:C) or lipopoly(I:C). The median of our results was reported by the horizontal line, the 25 and 75 interquartile range by the box and the minimum and the maximum by the whiskers. *: *p* < 0.05 versus the other group

At exacerbation and steady state, V + V+ cDC and monocytes expressed significantly more TLR3 than those of V + V- patients (Fig. 5a-b), but not MDA5 [see Additional file 7]. At exacerbation, the RIGI expression was higher in cDC and pDC but not in monocytes from V + V+ patients (*p* < 0.05) (Fig. 5a-b). There was no difference for CD86 and HLA-DR [see Additional file 7].

Secretion of cytokines by unstimulated PBMC was similar in V + V- and V + V+ patients at both times [see Additional file 6]. The production of IL-29 in response to gardiquimod was significantly lower in PBMC of V + V+ patients during exacerbation (*p* < 0.05) [see Additional file 4]. IFN-γ secretion in response to poly(I:C) or lipopoly(I:C) was also significantly decreased (Fig. 5c). At steady state, the same trend was observed for lipopoly(I:C)-induced production of IL-29 (Fig. 5c, *p* < 0.05) and IFN-γ (p = NS) [see Additional file 6]. The levels of the other cytokines did not differ between groups.

Compared to steady state, IFN-γ levels were significantly lower in poly(I:C)-stimulated PBMC at exacerbation in V + V- patients but not in V + V+ patients [see Additional file 6]. The same was observed for IL-1β production in gardiquimod-stimulated PBMC (*p* < 0.05).

## Discussion

This study was designed to assess the alteration of virus sensors and its association with impaired innate immune response during virus-induced asthma exacerbation in children. To test our hypothesis, patients were their own control and were analyzed according to their viral status. We further focused our work on a previously described subgroup prone to viral re-infection (V + V+) [15]. Each patient was evaluated during exacerbation and at steady state to distinguish the inflammation induced by exacerbation from the one due to asthma itself, which was done in very few other studies [27, 28].

Exacerbations were triggered by a virus in 62% of the children. Among them, 80% were hRV and type C was the most commonly identified [29]. We first observed that the clinical characteristics of the different groups of patients were not different at both times.

We have shown that IFN-β and IL-29 levels in V+ did not differ from those in V- patients. Low production of type I and III interferons by AEC and alveolar macrophages experimentally infected by hRV has been reported in asthmatic children and adults [8–10, 14], more profound in severe atopic asthma [12]. In the present study, patients infected with hRV appeared to display the same impairment (data not shown). In contrast, Bergauer et al. recently reported that IFN-α levels were markedly increased in a small cohort of hRV-infected symptomatic asthmatic children (4.8 ± 0.64 years) as compared with infected but asymptomatic patients whereas IL-29 synthesis remains unchanged [11]. Our results suggest that IFN-β and IFN-λ production is altered during virus-induced exacerbations in asthmatic children. Compared to our study, the population differs from that of Bergauer et al. by the age and also the severity of the exacerbation [11]. Furthermore, their results seems to be inconsistent on a larger cohort. At last in our V+ patients, the alteration of the anti-viral response was not linked to a modification of the expression of PRR of blood DC and monocytes nor with a blockade of their function. These results might also suggest that the alteration of the anti-viral response might preferentially be observed in the airways compared with blood cells.

The antiviral response was further analyzed among the V+ patients. We observed that V + V+ patients, infected at both times, produced lower levels of IFN-γ than V + V- patients during exacerbation. IL-29 and IFN-γ production were also lower at steady state, despite the presence of a new virus. As compared with V + V-, TLR3 and RNA-helicases were overexpressed on circulating APCs in V + V+ patients. During exacerbation, their PBMC showed an altered IFN-γ and IL-29 production after TLR3 and RIGI activation, that might facilitate the viral re-infection according to the IL-29 function [30]. The IL-29 production in response to TLR7-8 ligand was also impaired, suggesting a defective function of these receptors in PBMC from V + V+ patients, as recently reported in alveolar macrophages [14]. IFN-γ and IL-29 production impairment may be due to an altered signaling. Different mechanisms might be responsible for this defective production. Transforming growth factor-β inhibits IFN production in response to hRV [31]. The implication of suppressor of cytokine signaling (SOCS)1 overexpression in airway epithelium of severe asthmatic children have been suggested [32] although this is controversial [33]. Our data suggest that a specific alteration of the anti-viral response related to a dysfunction of virus sensors characterize these re-infected patients.

Viral infection during the exacerbation has also an impact on the airway inflammation as shown by the persistent increased sputum eosinophilia and its correlation with the IL5 concentrations, in agreement with Norzila et al. [27]. Th2 cytokines alter the innate immune response to viral infection and favor the development of a specific inflammatory reaction [34]. In V + V+, the lack of increase in IL-5 levels suggests that virus-infections don’t directly influence IL-5 in this subgroup. Nevertheless, it has been reported that viral infection in an allergic environment can induce IL-5 synthesis by CD8^+^ T cells, probably due to PRR activation in DC [35]. Interestingly, the production of IL-5 after TLR7-8 stimulation was primed in PBMC from V+ patients, a result probably due to the V + V- subgroup. Concomitantly, DC activation during exacerbation was demonstrated by the over-expression of CD86 and contributes to the APC propensity to induce IL-5 secretion by T lymphocytes in infected patients. The link between Th2 inflammation and antiviral response is also illustrated by the restoration of IFN-α production by plasmacytoid DC in allergic asthmatic children treated with omalizumab [36].

In re-infected patients, the inflammatory reaction was characterized by a strong secretion of IL-1β, which might be involved in the neutrophil recruitment observed at steady state. This feature was also associated with the production of IL-22, which promotes smooth muscle cells proliferation [37]. Interestingly, Simpson et al. reported that adult asthmatic with a neutrophilic inflammation and a high level of IL-1β in sputum have a reduced ability to produce IFN-α in response to hRV [38]. We suggest a link between this population and our group of V + V+ children. Long term follow-up is needed to define if the specificities of inflammation are mostly related to the repeated viral infection.

## Conclusion

Our results support a more pronounced defect in IFN-γ and IFN-λ secretion during virus-triggered exacerbation in the asthmatic children prone to viral re-infection. This defect is associated with an overexpression of virus sensors, a defective response to the corresponding ligands and with a specific airway inflammation. The benefit of strategies integrating an antiviral approach in this subgroup of patients should be further explored.

## Additional files

Additional file 1: Gating strategy for the analysis of conventionnal and plasmacytoid dendritic cell (cDC and pDC, respectively) in peripheral blood mononuclear cells (PBMC) from asthmatic children. (PDF 300 kb)

Additional file 2: Characteristics of the exacerbation at inclusion in the overall population, and comparison according to the viral status. (PDF 262 kb)

Additional file 3: Repartition of the exacerbations during the year according to the viral status. a) According to the viral status at the exacerbation. b) According to viral status at steady state in virus infected patients at the exacerbation. (PDF 353 kb)

Additional file 4: Concentrations of cytokines in asthmatic patients by viral status during the exacerbation and at steady state. Cytokines concentrations were measured during exacerbation (upper part) or at steady state (lower part) in plasma, sputum fluids and supernatants of MNC stimulated with Poly(I:C), Gardiquimod, lipopoly(I:C) or not (Medium). Patients infected by virus (V+) or not infected (V-) during the exacerbation were compared. Results are expressed as pg/ml (median with interquartile range [IQR]). ND: not detectable, NE: Not evaluated. (PDF 555 kb)

Additional file 5: Phenotype of blood antigen-presenting cells from asthmatic children by viral status during the exacerbation and at steady state. The upper part reported data collected during exacerbation from infected (V+) or not infected (V-) patients during the exacerbation, whereas the lower part showed the data obtained at steady state. The phenotype was analyzed in conventional and plasmacytoid DC (cDC and pDC, respectively) as well as in monocytes during the exacerbation and at steady state, respectively. Results are expressed as median of fluorescence intensity (MFI) with interquartile range [IQR]. ND: not detectable, NE: Not evaluated. (PDF 309 kb)

Additional file 6: Concentrations of cytokines in asthmatic patients prone to re-infection at steady state. Cytokines concentrations were measured during exacerbation or at steady state in plasma, sputum fluids and supernatants of MNC stimulated with Poly(I:C), Gardiquimod, lipopoly(I:C) or not (Medium). Patients only infected during the exacerbation (V + V-) were compared to those infected during both periods (V + V+). Results are expressed as pg/ml (median with interquartile range [IQR]). ND: not detectable, NE: Not evaluated. *:*p* < 0.05 significantly different from V- patients. (PDF 435 kb)

Additional file 7: Phenotype of blood antigen presenting cells in asthmatic patients prone to re-infection at steady state. Patients only infected during the exacerbation (V + V-) were compared to those infected during both periods (V + V+). The upper and the lower part showed the data collected in conventional and plasmacytoid DC (cDC and pDC, respectively) as well as in monocytes during the exacerbation and at steady state, respectively. Results are expressed as median of fluorescence intensity (MFI) with interquartile range [IQR]. (PDF 354 kb)

## Abbreviations

AEC: Airway epithelial cell
APC: Antigen-presenting cell
cDC: Conventionnal dendritic cell
CXCL: CXC chemokine ligand
DC: Dendritic cell
IFN: Interferon
IL: Interleukin
IQR: Interquartile range
MDA5: Melanoma Differentiation-Associated protein 5
PBMC: Peripheral blood mononuclear cell
pDC: Plasmacytoid DC
PRR: Pathogen recognition receptor
RIG-I: Retinoic Acid-Inducible Gene-I
Th2: T helper type 2
TLR: Toll like receptor
V-: Not-infected patients at exacerbation
V +: Virus infected patients at exacerbation
V + V-: Patients infected at exacerbation but not at steady state
V + V +: Patients infected both at exacerbation and steady state
