# Supplementary material for: Virus-triggered exacerbation in allergic asthmatic children: neutrophilic airway inflammation and alteration of virus sensors characterize a subgroup of patients
Source: Respir Res. 2017 Nov 14;18:191. doi: 10.1186/s12931-017-0672-0 (PMC5686805; doi:10.1186/s12931-017-0672-0)

Additionl file 1: Gating strategy for the analysis of conventionnal and plasmacytoid dendritic cell (cDC and pDC, respectively) in peripheral blood mononuclear cells (PBMC) from asthmatic children.

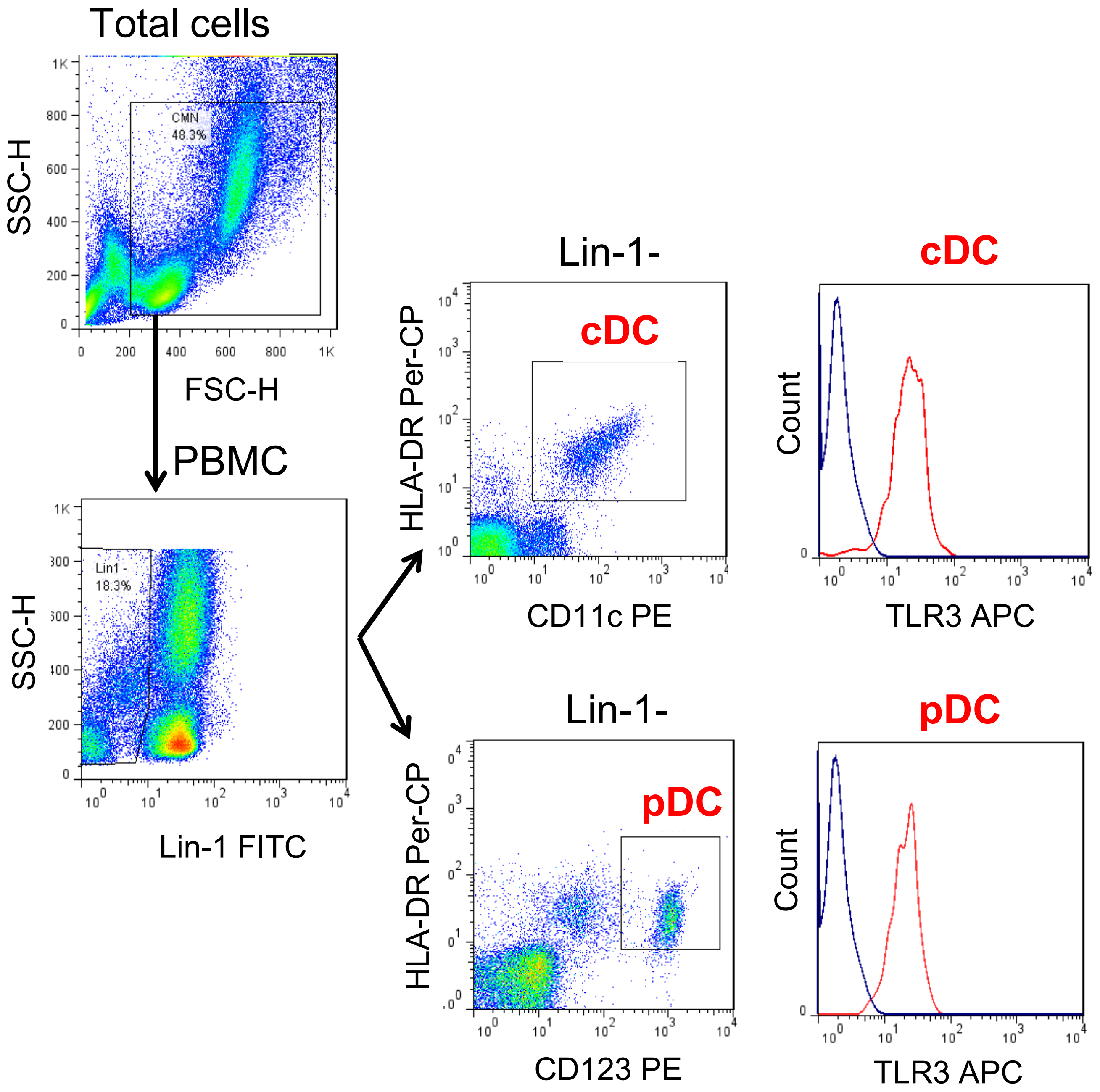

Supplement: Supplementary file 1 — Gating strategy for the analysis of conventionnal and plasmacytoid dendritic cell (cDC and pDC, respectively) in peripheral blood mononuclear cells (PBMC) from asthmatic children. (PDF 300 kb) [file 12931_2017_672_MOESM1_ESM.pdf]
