# Supplementary material for: Virus-triggered exacerbation in allergic asthmatic children: neutrophilic airway inflammation and alteration of virus sensors characterize a subgroup of patients
Source: Respir Res. 2017 Nov 14;18:191. doi: 10.1186/s12931-017-0672-0 (PMC5686805; doi:10.1186/s12931-017-0672-0)
Supplement: Supplementary file 2 — Characteristics of the exacerbation at inclusion in the overall population, and comparison according to the viral status. (PDF 262 kb) [file 12931_2017_672_MOESM2_ESM.pdf]

Additional file 2: Characteristics of the exacerbation at inclusion in the overall population and according to the viral status.

	All patients	V+	V-	V+V+	V+V-	p value* (ASD) V+ versus V-	p value* (ASD) V+V+ versus V+V-
**Median duration of hospitalization (days)**	4 [2-15]	4 [2-11]	5 [2-15]	4 [2-11]	4 [2-10]	0.11 (40.4)	0.72 (11.8)
**Median duration of oxygenotherapy (days)**	3 [0-10]	2 [0-10]	3 [0-9]	2 [0-10]	2 [0-9]	0.19 (33.0)	0.80 (8.1)

V+: identification of a virus (PCR) at the exacerbation, V-: no identification of a virus at the exacerbation, V+V+: identification of a virus at the exacerbation and at the steady state, V+V-: identification of a virus at the exacerbation but not at the steady state. Results are expressed as median with interquartile range between brackets. * Data were compared via the Mann-Whitney test. ASD: absolute standardized difference (%).
