# Supplementary material for: Virus-triggered exacerbation in allergic asthmatic children: neutrophilic airway inflammation and alteration of virus sensors characterize a subgroup of patients
Source: Respir Res. 2017 Nov 14;18:191. doi: 10.1186/s12931-017-0672-0 (PMC5686805; doi:10.1186/s12931-017-0672-0)
Supplement: Supplementary file 3 — Repartition of the exacerbations during the year according to the viral status. a) According to the viral status at the exacerbation. b) According to viral status at steady state in virus infected patients at the exacerbation. (PDF 353 kb) [file 12931_2017_672_MOESM3_ESM.pdf]

Additional File 3: Repartition of the exacerbations during the year according to the viral status. a) According to the viral status at the exacerbation. b) According to the viral status at steady state in virus infected patients at the exacerbation.

a)

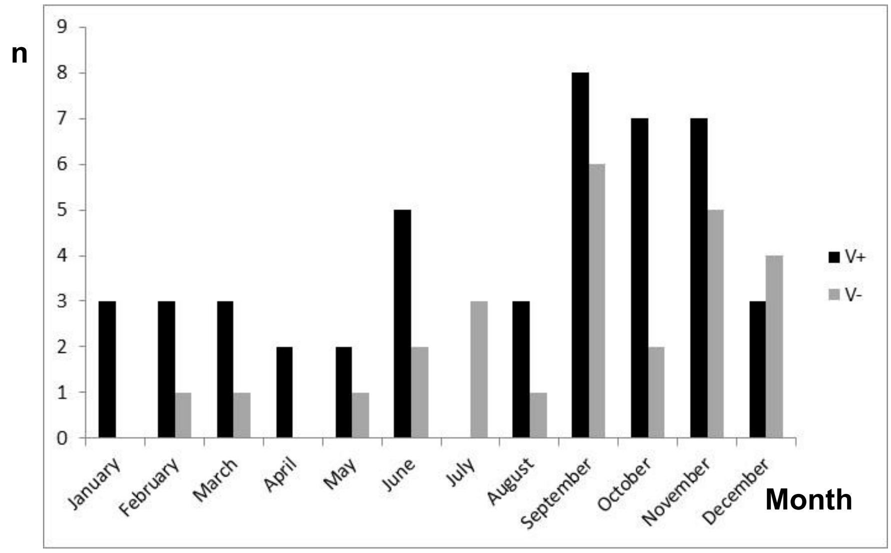

b)

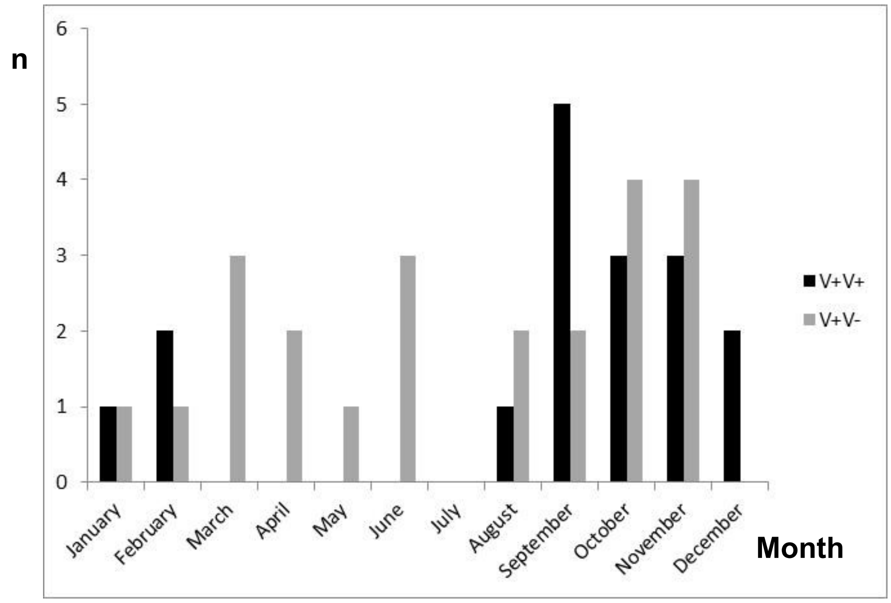

V+: identification of a virus (PCR) at inclusion, V-: no identification of a virus at inclusion, V+V+: identification of a virus at inclusion and at the steady state, V+V-: identification of a virus at inclusion but not at the steady state.
