# Supplementary material for: Virus-triggered exacerbation in allergic asthmatic children: neutrophilic airway inflammation and alteration of virus sensors characterize a subgroup of patients
Source: Respir Res. 2017 Nov 14;18:191. doi: 10.1186/s12931-017-0672-0 (PMC5686805; doi:10.1186/s12931-017-0672-0)
Supplement: Supplementary file 4 — Concentrations of cytokines in asthmatic patients by viral status during the exacerbation and at steady state. Cytokines concentrations were measured during exacerbation (upper part) or at steady state (lower part) in plasma, sputum fluids and supernatants of MNC stimulated with Poly(I:C), Gardiquimod, lipopoly(I:C) or not (Medium). Patients infected by virus (V+) or not infected (V-) during the exacerbation were compared. Results are expressed as pg/ml (median with interquartile range [IQR]). ND: not detectable, NE: Not evaluated. (PDF 555 kb) [file 12931_2017_672_MOESM4_ESM.pdf]

Additional file 4: Concentrations of cytokines in asthmatic patients according to their viral status at exacerbation. Cytokines concentrations were measured during exacerbation or at steady state in plasma, sputum fluids and supernatants of MNC stimulated with Poly(I:C), Gardiquimod, lipopoly(I:C) or not (Medium). Patients infected by virus (V+) or not infected (V-) during the exacerbation were compared. Results are expressed as pg/ml [median with interquartile range (IQR)]. ND: not detectable, NE: Not evaluated.

*: indicates a statistical significance between the 2 groups ($p < 0.05$). [1, 2, 3] indicate an absolute standardized difference greater than 0.2, 0.5 and 0.8 respectively

	**Exacerbation**	Serum		Sputum		MNC Medium		MNC P(I:C)		MNC Gardiquimod		MNC Lipopoly(I:C)	
		**V-**	**V+**	**V-**	**V+**	**V-**	**V+**	**V-**	**V+**	**V-**	**V+**	**V-**	**V+**
CXCL8	**Median**	**ND**	**ND**	**1494**	**2725** [1]	**2384**	**2407**	**5180**	**2325**	**33418**	**31516**	**4106**	**4145**
	[IQR]			*[627-5078]*	*[954-7685]*	*[620-10672]*	*[509-3332]*	*[1472-13355]*	*[1560-28034]*	*[5563-51628]*	*[2110-63756]*	*[1967-20323]*	*[1650-17323]*
IFN-β	**Median**	**33**	**37**	**5**	**16** [1]	**17**	**5** [1]	**5**	**5** [1]	**5**	**5**	**NE**	**NE**
	[IQR]	*[21-93]*	*[21-91]*	*[5-19]*	*[5-23]*	*[5-30]*	*[5-20]*	*[5-27]*	*[5-20]*	*[5-20]*	*[5-19]*		
IFN-γ	**Median**	**352**	**362**	**2**	**2** [1]	**2**	**2**	**78**	**80**	**60**	**44**	**216**	**69** [1]
	[IQR]	*[108-725]*	*[86-935]*	*[1-37]*	*[2-61]*	*[2-38]*	*[2-25]*	*[24-199]*	*[22-181]*	*[2-353]*	*[14-229]*	*[34-741]*	*[42-169]*
IL-1β	**Median**	**2**	**2**	**45**	**37**	**2**	**15 *** [1]	**4**	**2**	**86**	**221 *** [1]	**14**	**2** [1]
	[IQR]	*[2-14]*	*[2-78]*	*[2-160]*	*[2-60]*	*[2-10]*	*[2-52]*	*[2-31]*	*[2-49]*	*[9-219]*	*[35-878]*	*[2-111]*	*[2-64]*
IL-22	**Median**	**117**	**93** [1]	**119**	**98**	**2**	**54** [1]	**105**	**79** [1]	**125**	**70 *** [2]	**110**	**70** [1]
	[IQR]	*[67-171]*	*[31-161]*	*[6-416]*	*[5-316]*	*[47-182]*	*[10-98]*	*[38-256]*	*[10-103]*	*[83-174]*	*[15-106]*	*[71-150]*	*[23-114]*
IL-29	**Median**	**640**	**614**	**5**	**7**	**7**	**19** [1]	**5**	**12** [1]	**5**	**10 *** [1]	**12**	**12**
	[IQR]	*[388-1181]*	*[253-1141]*	*[5-47]*	*[5-36]*	*[5-18]*	*[5-35]*	*[5-24]*	*[5-36]*	*[5-23]*	*[5-37]*	*[5-40]*	*[5-27]*
IL-5	**Median**	**41**	**40**	**13**	**57 *** [1]	**2**	**2** [1]	**2**	**7** [2]	**2**	**10 *** [1]	**4**	**3**
	[IQR]	*[15-74]*	*[6-85]*	*[2-61]*	*[4-215]*	*[2-3]*	*[2-17]*	*[2-6]*	*[2-36]*	*[2-14]*	*[2-24]*	*[2-16]*	*[2-24]*
IL-6	**Median**	**2**	**2**	**25**	**21** [1]	**16**	**66**	**179**	**169**	**2463**	**1130**	**318**	**292**
	[IQR]	*[2-13]*	*[2-16]*	*[5-35]*	*[2-86]*	*[2-229]*	*[2-246]*	*[37-342]*	*[41-380]*	*[431-4949]*	*[450-6573]*	*[72-870]*	*[37-960]*

	**Steady State**	Serum		Sputum		MNC Medium		MNC P(I:C)		MNC Gardiquimod		MNC Lipopoly(I:C)	
		**V-**	**V+**	**V-**	**V+**	**V-**	**V+**	**V-**	**V+**	**V-**	**V+**	**V-**	**V+**
CXCL8	**Median**	**ND**	**ND**	**761**	**135** [2]	**3823**	**5425**	**4539**	**11521** [1]	**20952**	**19084**	**5880**	**10358**
	[IQR]			*[239-2512]*	*[2-828]*	*[899-24635]*	*[835-24814]*	*[605-20981]*	*[1132-45332]*	*[8538-41209]*	*[10626-32515]*	*[2932-16074]*	*[2330-17301]*
IFN-β	**Median**	**44**	**39**	**5**	**5**	**5**	**5** [1]	**5**	**7**	**5**	**5**	**NE**	**NE**
	[IQR]	*[21-84]*	*[5-143]*	*[5-31]*	*[5-22]*	*[5-21]*	*[5-18]*	*[5-19]*	*[5-18]*	*[5-20]*	*[5-21]*		
IFN-γ	**Median**	**320**	**148** [1]	**2**	**2**	**38**	**41**	**92**	**343 *** [1]	**603**	**611** [1]	**771**	**3605**
	[IQR]	*[113-806]*	*[47-756]*	*[2-31]*	*[2-10]*	*[6-88]*	*[2-214]*	*[34-437]*	*[48-6664]*	*[130-4230]*	*[313-9901]*	*[112-9958]*	*[314-11002]*
IL-1β	**Median**	**2**	**10** [1]	**7**	**45**	**19**	**51 *** [1]	**28**	**60 *** [2]	**297**	**315** [1]	**53**	**71**
	[IQR]	*[2-24]*	*[2-67]*	*[2-206]*	*[2-208]*	*[2-60]*	*[5-119]*	*[2-63]*	*[15-243]*	*[160-503]*	*[188-1353]*	*[24-190]*	*[17-285]*
IL-22	**Median**	**142**	**95 *** [2]	**96**	**23** [1]	**148**	**121**	**137**	**119** [1]	**109**	**108**	**117**	**113**
	[IQR]	*[87-175]*	*[17-144]*	*[8-686]*	*[5-542]*	*[65-262]*	*[80-154]*	*[94-201]*	*[66-228]*	*[71-202]*	*[65-172]*	*[78-160]*	*[84-155]*
IL-29	**Median**	**782**	**717**	**104**	**49**	**19**	**33** [1]	**20**	**29**	**10**	**26 *** [2]	**20**	**10**
	[IQR]	*[441-1026]*	*[292-1361]*	*[10-198]*	*[10-172]*	*[10-36]*	*[10-52]*	*[10-49]*	*[10-81]*	*[10-27]*	*[10-39]*	*[10-63]*	*[10-57]*
IL-5	**Median**	**30**	**36**	**3**	**31 *** [2]	**1**	**13 *** [2]	**1**	**5** [1]	**1**	**14** [1]	**1**	**2**
	[IQR]	*[24-69]*	*[10-96]*	*[1-41]*	*[14-279]*	*[1-11]*	*[1-42]*	*[1-14]*	*[1-41]*	*[1-29]*	*[1-48]*	*[1-19]*	*[1-17]*
IL-6	**Median**	**2**	**2**	**8**	**2** [1]	**153**	**377**	**404**	**796 *** [1]	**5574**	**3557** [1]	**986**	**728**
	[IQR]	*[1-5]*	*[1-2]*	*[1-14]*	*[1-2]*	*[8-668]*	*[8-714]*	*[88-1723]*	*[257-2652]*	*[3393-6893]*	*[1103-7081]*	*[121-2520]*	*[346-2945]*
