# Supplementary material for: Virus-triggered exacerbation in allergic asthmatic children: neutrophilic airway inflammation and alteration of virus sensors characterize a subgroup of patients
Source: Respir Res. 2017 Nov 14;18:191. doi: 10.1186/s12931-017-0672-0 (PMC5686805; doi:10.1186/s12931-017-0672-0)
Supplement: Supplementary file 5 — Phenotype of blood antigen-presenting cells from asthmatic children by viral status during the exacerbation and at steady state. The upper part reported data collected during exacerbation from infected (V+) or not infected (V-) patients during the exacerbation, whereas the lower part showed the data obtained at steady state. The phenotype was analyzed in conventional and plasmacytoid DC (cDC and pDC, respectively) as well as in monocytes during the exacerbation and at steady state, respectively. Results are expressed as median of fluorescence intensity (MFI) with interquartile range [IQR]. ND: not detectable, NE: Not evaluated. (PDF 309 kb) [file 12931_2017_672_MOESM5_ESM.pdf]

Additional file 5: Phenotype of blood antigen-presenting cells from asthmatic children according to their viral status at exacerbation. The upper part reported data collected during exacerbation from infected (V+) or not infected (V-) patients during the exacerbation, whereas the lower part showed the data obtained at steady state. The phenotype was analyzed in conventional and plasmacytoid DC (cDC and pDC, respectively) as well as in monocytes during the exacerbation and at steady state, respectively. Results are expressed as median of fluorescence intensity (MFI) [interquartile range (IQR)]. ND: not detectable, NE: Not evaluated.

*: indicates a statistical significance between the 2 groups ($p < 0.05$) . [1, 2, 3] indicate an absolute standardized difference greater than 0.2, 0.5 and 0.8 respectively

	**Exacerbation**	cDC		pDC		Monocyte	
		**V-**	**V+**	**V-**	**V+**	**V-**	**V+**
CD86	**Median**	**7**	**10 *[1]**	**5**	**7[1]**	**6**	**8 *[2]**
	[IQR]	*[5-11]*	*[6-14]*	*[3-13]*	*[4-21]*	*[5-7]*	*[6-12]*
HLA-DR	**Median**	**23**	**38[1]**	**20**	**31[1]**	**13**	**16[1]**
	[IQR]	*[18-63]*	*[20-81]*	*[12-38]*	*[13-81]*	*[9-38]*	*[11-42]*
TLR3	**Median**	**29**	**33**	**38**	**37**	**75**	**82**
	[IQR]	*[23-54]*	*[18-64]*	*[21-54}*	*[22-62]*	*[41-134]*	*[53-144]*
RIGI	**Median**	**50**	**64[1]**	**51**	**60**	**102**	**113**
	[IQR]	*[20-78]*	*[35-100]*	*[20-99]*	*[38-71]*	*[48-319]*	*[63-165]*
MDA5	**Median**	**51**	**68**	**32**	**38**	**81**	**92**
	[IQR]	*[32-103]*	*[33-92]*	*[17-61]*	*[19-71]*	*[43-143]*	*[58-171]*

	**Steady State**	cDC		pDC		Monocyte	
		**V-**	**V+**	**V-**	**V+**	**V-**	**V+**
CD86	**Median**	**6**	**10[1]**	**6**	**7 *[1]**	**6**	**9[2]**
	[IQR]	*[5-12]*	*[6-14]*	*[4-12]*	*[5-24]*	*[5-10]*	*[6-13]*
HLA-DR	**Median**	**44**	**39**	**63**	**25[1]**	**25**	**31**
	[IQR]	*[26-150]*	*[30-73]*	*[19-125]*	*[12-74]*	*[16-74]*	*[18-54]*
TLR3	**Median**	**42**	**41**	**44**	**41**	**72**	**90[1]**
	[IQR]	*[24-61]*	*[19-62]*	*[28-63]*	*[29-60]*	*[53-119]*	*[72-135]*
RIGI	**Median**	**53**	**63**	**39**	**44**	**74**	**96[1]**
	[IQR]	*[26-70]*	*[32-85]*	*[23-57]*	*[27-70]*	*[59-198]*	*[73-156]*
MDA5	**Median**	**49**	**53**	**31**	**38**	**138**	**79[1]**
	[IQR]	*[18-91]*	*[26-86]*	*[17-50]*	*[17-72]*	*[55-182]*	*[52-151]*
