# Supplementary material for: Virus-triggered exacerbation in allergic asthmatic children: neutrophilic airway inflammation and alteration of virus sensors characterize a subgroup of patients
Source: Respir Res. 2017 Nov 14;18:191. doi: 10.1186/s12931-017-0672-0 (PMC5686805; doi:10.1186/s12931-017-0672-0)
Supplement: Supplementary file 6 — Concentrations of cytokines in asthmatic patients prone to re-infection at steady state. Cytokines concentrations were measured during exacerbation or at steady state in plasma, sputum fluids and supernatants of MNC stimulated with Poly(I:C), Gardiquimod, lipopoly(I:C) or not (Medium). Patients only infected during the exacerbation (V + V-) were compared to those infected during both periods (V + V+). Results are expressed as pg/ml (median with interquartile range [IQR]). ND: not detectable, NE: Not evaluated. *:p < 0.05 significantly different from V- patients. (PDF 435 kb) [file 12931_2017_672_MOESM6_ESM.pdf]

Additional file 6: Concentrations of cytokines in asthmatic patients prone to re-infection at steady state. Cytokines concentrations were measured during exacerbation or at steady state in plasma, sputum fluids and supernatants of MNC stimulated with Poly(I:C), Gardiquimod, lipopoly(I:C) or not (Medium). Patients only infected during the exacerbation (V+V-) were compared to those infected during both periods (V+V+). Results are expressed as pg/ml [median with interquartile range (IQR)]. ND :not detectable, NE: Not evaluated. *: indicates a statistical significance (p <0.0) between the 2 groups. [1, 2, 3] indicate an absolute standardized difference greater than 0.2, 0.5 and 0.8 respectively

	**Exacerbation**	Serum		Sputum		MNC Medium		MNC P(I:C)		MNC Gardiquimod		MNC Lipopoly(I:C)	
		**V+ V-**	**V+ V+**	**V+ V-**	**V+ V+**	**V+ V-**	**V+ V+**	**V+ V-**	**V+ V+**	**V+ V-**	**V+ V+**	**V+ V-**	**V+ V+**
**CXCL8**	**Median**	**ND**	**ND**	**3929**	**2029 [1]**	**5543**	**2057 [1]**	**8804**	**2856 [1]**	**36749**	**30647**	**8065**	**3895**
	[IQR]			*[1188-7259]*	*[710-5251]*	*[509-30823]*	*[893-8093]*	*[1575-32237]*	*[1560-13529]*	*[2110-70279]*	*[6027-61665]*	*[2359-21575]*	*[1971-16866]*
**IFN-β**	**Median**	**42**	**37**	**16**	**5**	**5**	**17 [2]**	**5**	**18 [1]**	**5**	**17 [1]**	**NE**	**NE**
	[IQR]	*[23-91]*	*[19-101]*	*[5-21]*	*[5-34]*	*[5-16]*	*[5-22]*	*[5-15]*	*[5-36]*	*[5-16]*	*[5-32]*		
**IFN-γ**	**Median**	**493**	**207 * [2]**	**12**	**1 * [3]**	**2**	**14**	**95**	**27 * [2]**	**47**	**28 [1]**	**82**	**32 * [3]**
	[IQR]	*[104-935]*	*[28-499]*	*[2-109]*	*[1-2]*	*[2-35]*	*[2-25]*	*[15-322]*	*[15-45]*	*[23-200]*	*[2-192]*	*[57-243]*	*[2-76]*
**IL-1β**	**Median**	**2**	**2**	**4**	**58 * [2]**	**31**	**7 [1]**	**2**	**2**	**204**	**260**	**2**	**2**
	[IQR]	*[1-134]*	*[2-18]*	*[2-70]*	*[39-225]*	*[2-60]*	*[2-24]*	*[2-9]*	*[2-48]*	*[65-878]*	*[10-673]*	*[2- 64]*	*[2-85]*
**IL-22**	**Median**	**62**	**78**	**103**	**78**	**42**	**69**	**79**	**64**	**65**	**72 [1]**	**70**	**78**
	[IQR]	*[10-142]*	*[30-137]*	*[5-491]*	*[10-123]*	*[10-107]*	*[16-96]*	*[10-116]*	*[15-99]*	*[15-276]*	*[15-89]*	*[26-114]*	*[17-99]*
**IL-29**	**Median**	**444**	**895 * [1]**	**8**	**12**	**15.5**	**33 [1]**	**10**	**12**	**15**	**10 ***	**8**	**11**
	[IQR]	*[277-944]*	*[248-1550]*	*[5-36]*	*[3-49]*	*[5-40]*	*[5-34]*	*[5-45]*	*[5-34]*	*[5-40]*	*[5-37]*	*[5-31]*	*[5-19]*
**IL-5**	**Median**	**48**	**17.5 [1]**	**95**	**13 * [2]**	**2**	**4 [2]**	**3**	**13**	**6**	**13**	**2**	**8**
	[IQR]	*[6-89]*	*[5-73]*	*[8-227]*	*[4-83]*	*[2-7]*	*[2-17]*	*[2-42]*	*[2-32]*	*[2-37]*	*[2-13]*	*[2-42]*	*[2-18]*
**IL-6**	**Median**	**2**	**10 * [2]**	**23**	**61 [1]**	**163**	**29 [1]**	**256**	**111**	**1128**	**1528**	**330**	**219**
	[IQR]	*[2-9]*	*[2-27]*	*[2-83]*	*[8-334]*	*[2-318]*	*[2-225]*	*[16-470]*	*[48-368]*	*[498-7690]*	*[431-4929]*	*[116-784]*	*[37-1133]*

	**Steady State**	Serum		Sputum		MNC Medium		MNC P(I:C)		MNC Gardiquimod		MNC Lipopoly(I:C)	
		**V+ V-**	**V+V+**	**V+ V-**	**V+ V+**	**V+ V-**	**V+ V+**	**V+ V-**	**V+ V+**	**V+ V-**	**V+ V+**	**V+ V-**	**V+ V+**
**CXCL8**	**Median**	**ND**	**ND**	**178**	**124 [1]**	**4808**	**7444**	**11521**	**12280**	**19259**	**18924**	**10366**	**10458 [1]**
	[IQR]			*[2-612]*	*[10-43724]*	*[898-34261*	*[1719-19284]*	*[1243-41809]*	*[1841-48855]*	*[10742-41259]*	*[11335-30474]*	*[4262-17339]*	*[2245-16074]*
**IFN-β**	**Median**	**48**	**39**	**19**	**5 [3]**	**5**	**16**	**5**	**16 [1]**	**5**	**19 [1]**	**NE**	**NE**
	[IQR]	*[21-114]*	*[5-141]*	*[5-60]*	*[5-6]*	*[5-22]*	*[5-20]*	*[5-25]*	*[5-19]*	*[5-22]*	*[5-26]*		
**IFN-γ**	**Median**	**172**	**119**	**2**	**2 [1]**	**32**	**58 [1]**	**343**	**300**	**547**	**620**	**3605**	**370 [1]**
	[IQR]	*[21-944]*	*[76-641]*	*[2-18]*	*[2-10]*	*[2-135]*	*[6-166]*	*[50-2689]*	*[29-7625]*	*[314-8661]*	*[238-2335]*	*[554-10551]*	*[123-11389]*
**IL-1β**	**Median**	**2**	**10**	**31**	**231 [2]**	**64**	**14 [1]**	**52**	**124 [1]**	**215**	**656 [1]**	**72**	**24 [1]**
	[IQR]	*[1-89]*	*[2-35]*	*[2-87]*	*[2-519]*	*[5-119]*	*[4-220]*	*[8-185]*	*[17-403]*	*[162-2506]*	*[228-1353]*	*[32-285]*	*[7-311]*
**IL-22**	**Median**	**95**	**124 [1]**	**5**	**542 * [3]**	**101**	**125 [1]**	**110**	**116**	**140**	**101 [1]**	**112**	**112**
	[IQR]	*[8-146]*	*[22-142]*	*[5-6]*	*[40-720]*	*[75-152]*	*[80-154]*	*[63-175]*	*[69-228]*	*[75-206]*	*[65-161]*	*[105-150]*	*[78-258]*
**IL-29**	**Median**	**663**	**713**	**139**	**14 [2]**	**33**	**35 [1]**	**36**	**18**	**27**	**20**	**37**	**10* [2]**
	[IQR]	*[278-1376]*	*[343-1352]*	*[16-547]*	*[10-70]*	*[10-42]*	*[10-77]*	*[10-105]*	*[10-89]*	*[10-53]*	*[10-39]*	*[10-78]*	*[7-40]*
**IL-5**	**Median**	**36**	**32**	**149**	**34 [1]**	**7**	**14**	**3**	**8**	**7**	**15**	**3**	**1 [1]**
	[IQR]	*[12-75]*	*[6-147]*	*[10-292]*	*[28-73]*	*[1-86]*	*[1-17]*	*[1-58]*	*[1-15]*	*[1-53]*	*[1-46]*	*[1-51]*	*[1-15]*
**IL-6**	**Median**	**2**	**2 [2]**	**1**	**2 [2]**	**379**	**370**	**796**	**1453**	**2963**	**3712**	**698**	**1148**
	[IQR]	*[1-4]*	*[2-5]*	*[1-2]*	*[1-73]*	*[8-1354]*	*[4-577]*	*[209-2616]*	*[247-4252]*	*[1695-6808]*	*[780-9376]*	*[326-2945]*	*[330-5113]*
