# Supplementary material for: Virus-triggered exacerbation in allergic asthmatic children: neutrophilic airway inflammation and alteration of virus sensors characterize a subgroup of patients
Source: Respir Res. 2017 Nov 14;18:191. doi: 10.1186/s12931-017-0672-0 (PMC5686805; doi:10.1186/s12931-017-0672-0)
Supplement: Supplementary file 7 — Phenotype of blood antigen presenting cells in asthmatic patients prone to re-infection at steady state. Patients only infected during the exacerbation (V + V-) were compared to those infected during both periods (V + V+). The upper and the lower part showed the data collected in conventional and plasmacytoid DC (cDC and pDC, respectively) as well as in monocytes during the exacerbation and at steady state, respectively. Results are expressed as median of fluorescence intensity (MFI) with interquartile range [IQR]. (PDF 354 kb) [file 12931_2017_672_MOESM7_ESM.pdf]

Additional file 7: Phenotype of blood antigen presenting cells in asthmatic patients prone to re-infection at steady state. Patients only infected during the exacerbation (V+V-) were compared to those infected during both periods (V+V+). The upper and the lower parts show the data collected in conventional and plasmacytoid DC (cDC and pDC, respectively) as well as in monocytes during the exacerbation and at steady state, respectively. Results are expressed as median of fluorescence intensity (MFI) [interquartile range (IQR)].

*: indicates a statistical significance between the 2 groups ($p < 0.05$) . [1, 2, 3] indicate an absolute standardized difference greater than 0.2, 0.5 and 0.8 respectively

	**Exacerbation**	cDC		pDC		Monocyte	
		**V+ V-**	**V+ V+**	**V+ V-**	**V+ V+**	**V+ V-**	**V+ V+**
CD86	**Median**	**9**	**10**	**9**	**6** [1]	**9**	**9**
	[IQR]	*[5-15]*	*[6-13]*	*[5-26]*	*[4-21]*	*[5-13]*	*[6-12]*
HLA-DR	**Median**	**29**	**40** [1]	**21**	**42** [1]	**14**	**23** [1]
	[IQR]	*[14-66]*	*[21-86]*	*[11-47]*	*[9-109]*	*[9-31]*	*[11-42]*
TLR3	**Median**	**25**	**53 *** [2]	**31**	**48 *** [3]	**59**	**111 *** [3]
	[Interquartile range]	*[17-38]*	*[22-123]*	*[15-41]*	*[36-85]*	*[38-84]*	*[65-175]*
RIGI	**Median**	**60**	**74** [1]	**55**	**64 *** [2]	**75**	**130** [2]
	[IQR]	*[35-90]*	*[40-200]*	*[31-63]*	*[40-97]*	*[49-126]*	*[95-150]*
MDA5	**Median**	**48**	**74** [1]	**34**	**60** [1]	**69**	**102** [2]
	[IQR]	*[17-84]*	*[50-92]*	*[11-61]*	*[11-80]*	*[31-102]*	*[59-404]*

	**Steady State**	cDC		pDC		Monocyte	
		**V+ V-**	**V+ V+**	**V+ V-**	**V+ V+**	**V+ V-**	**V+ V+**
CD86	**Median**	**10**	**8**	**9**	**6**	**12**	**9**
	[IQR]	*[7-13]*	*[5-16]*	*[5-25]*	*[4-28]*	*[6-13]*	*[6-14]*
HLA-DR	**Median**	**35**	**48** [1]	**21**	**26** [1]	**24**	**38** [2]
	[IQR]	*[29-48]*	*[33-86]*	*[11-42]*	*[13-101]*	*[17-42]*	*[1-70]*
TLR3	**Median**	**33**	**51** [2]	**39**	**48** [1]	**77**	**117 *** [3]
	[IQR]	*[18-50]*	*[24-75]*	*[23-49]*	*[31-60]*	*[64-90]*	*[80-198]*
RIGI	**Median**	**53**	**63**	**35**	**48**	**95**	**96**
	[IQR]	*[30-85]*	*[33-85]*	*[27-50]*	*[26-69]*	*[69-130]*	*[76-150]*
MDA5	**Median**	**43**	**54**	**28**	**43** [1]	**87**	**71** [1]
	[IQR]	*[26-83][*	*[28-79]*	*[15-42]*	*[25-72]*	*[59-134]*	*[53-168}*
